# The Adverse Effects of Androgen Deprivation Therapy in Prostate Cancer and the Benefits and Potential Anti-oncogenic Mechanisms of Progressive Resistance Training

**DOI:** 10.1186/s40798-020-0242-8

**Published:** 2020-02-13

**Authors:** Teresa Lam, Vita Birzniece, Mark McLean, Howard Gurney, Amy Hayden, Birinder S. Cheema

**Affiliations:** 1 School of Medicine, Western Sydney University, Penrith, NSW Australia; 2 Department of Diabetes and Endocrinology, Westmead Hospital, Westmead, NSW Australia; 3 Department of Diabetes and Endocrinology, Blacktown Hospital, Blacktown, NSW Australia; 4 School of Medicine, UNSW Sydney, Sydney, NSW Australia; 5 Garvan Institute of Medical Research, Darlinghurst, NSW Australia; 6 Translational Health Research Institute, Penrith, NSW Australia; 7 Crown Princess Mary Cancer Centre, Westmead, NSW Australia; 8 Department of Radiation Oncology, Blacktown Hospital, Blacktown, NSW Australia; 9 School of Science and Health, Western Sydney University, Penrith, NSW Australia

**Keywords:** Resistance training, Prostate cancer, Androgen deprivation therapy, Metabolic effects, Mitogenic pathways

## Abstract

Prostate cancer has the second highest incidence of all cancers amongst men worldwide. Androgen deprivation therapy (ADT) remains a common form of treatment. However, in reducing serum testosterone to castrate levels and rendering men hypogonadal, ADT contributes to a myriad of adverse effects which can affect prostate cancer prognosis. Physical activity is currently recommended as synergistic medicine in prostate cancer patients to alleviate the adverse effects of treatment. Progressive resistance training (PRT) is an anabolic exercise modality which may be of benefit in prostate cancer patients given its potency in maintaining and positively adapting skeletal muscle. However, currently, there is a scarcity of RCTs which have evaluated the use of isolated PRT in counteracting the adverse effects of prostate cancer treatment. Moreover, although physical activity in general has been found to reduce relapse rates and improve survival in prostate cancer, the precise anti-oncogenic effects of specific exercise modalities, including PRT, have not been fully established. Thus, the overall objective of this article is to provide a rationale for the in-depth investigation of PRT and its biological effects in men with prostate cancer on ADT. This will be achieved by (1) summarising the metabolic effects of ADT in patients with prostate cancer and its effect on prostate cancer progression and prognosis, (2) reviewing the existing evidence regarding the metabolic benefits of PRT in this cohort, (3) exploring the possible oncological pathways by which PRT can affect prostate cancer prognosis and progression and (4) outlining avenues for future research.

## Key Points

- Androgen deprivation therapy (ADT) is associated with adverse metabolic effects which can affect prognosis in men with prostate cancer.
- Progressive resistance training (PRT) is an exercise modality which can benefit both body composition and muscle function during ADT.
- PRT may exert its positive effects on prostate cancer prognosis through its modification of cancer signalling pathways.

## Introduction

Prostate cancer has the second highest incidence of all cancers amongst men worldwide and is the fifth leading cause of cancer death in men. In 2018, an estimated 1,276,106 new cases of prostate cancer was reported worldwide, with higher prevalence in the developed countries [1]. The mechanisms of prostate carcinogenesis have marked heterogeneity and consist of both genetic and environmental factors, with risk of the disease increasing with age and positive family history [2]. Although androgens (including testosterone and dihydrotestosterone) affect proliferation and differentiation of prostate luminal epithelium and drive prostate cancer cell growth, there are conflicting data on the role of endogenous testosterone in human prostate cancer pathogenesis de novo. A pooled analysis of 18 prospective studies showed no association between the risk of prostate cancer and testosterone levels [3]. Yet, positive associations have been found between mutations in genes involved in the biosynthesis and degradation of testosterone, and higher prostate cancer risk [4, 5]. Although similar controversies occur regarding the link between obesity, diabetes and risk of prostate cancer development [6–10], there is now strong evidence that being overweight or obese increases the risk of advanced prostate cancer [11].

Androgen receptor signalling strongly promotes growth, proliferation and invasiveness of prostate cancer. Thus, androgen deprivation therapy (ADT), using gonadotrophin-releasing hormone (GnRH) analogues and/or anti-androgen agents, is a common and effective therapy for patients with locally advanced and metastatic prostate cancer. Long-acting GnRH analogues, such as leuprolide, cause downregulation of the pituitary-gonadal axis, resulting in ‘chemical castration’ due to suppression of testicular testosterone production. ADT leads to a decline of prostate-specific antigen (PSA) in about 90% of patients [12]. However, in rendering the patient severely hypogonadal, ADT is associated with significant adverse metabolic effects. Consequences of ADT include the development of insulin resistance, reduced muscle and bone mineral density (BMD), increased fat mass, sexual dysfunction and reduced quality of life [13, 14]. Thus, there is a need for secondary treatment methods to combat the adverse effects of ADT.

Progressive resistance training (PRT) is an anabolic form of exercise that involves challenging the skeletal muscles with unaccustomed loads through use of free weights (e.g. barbells, dumbbells, medicine balls, sandbags), machine weights (e.g. leg press) and/or body weight (e.g. push-ups, pull-ups) and impact loading/plyometric exercises such as jumping. To facilitate continued muscular anabolic adaptation over the long-term, training variables including intensity and volume must be manipulated over time [15]. It is well established that PRT can treat sarcopenia in older men and women (over 50 years) [16] and muscle wasting in some chronic diseases, including patients affected with end-stage renal disease [17] and AIDS-related muscle wasting [18]. The myogenic effect of PRT has been associated with many other beneficial physiological, functional and psychological adaptations across a range of healthy and chronically diseased populations. The benefits are likely to extend to patients with cancer. In fact, the Clinical Oncology Society of Australia has recently endorsed the use of PRT as standard practice in cancer care [19].

To date, only a few robust studies have investigated the efficacy of PRT in patients receiving ADT for prostate cancer and the specific biological effects of this exercise modality are not completely understood in this cohort. Therefore, the overall objective of this review paper is to provide a rationale for the in-depth investigation of PRT and its biological effects in men with prostate cancer on ADT. This will be achieved by (1) summarising the adverse consequences of ADT in patients with prostate cancer and its effect on prostate cancer progression and prognosis, (2) summarising the existing evidence regarding the benefits of PRT in this cohort, (3) exploring the possible oncological pathways by which PRT can affect prostate cancer prognosis and progression and (4) outlining avenues for future research.

## Adverse Consequences of Androgen Deprivation Therapy and Their Potential Effects on Prostate Cancer Progression and Prognosis

Androgens play a vital role in the regulation of body composition, insulin and glucose sensitivity, growth factors and inflammation. Thus, the development of hypogonadism following ADT is associated with multiple adverse effects which have potential negative effects on prostate cancer prognosis.

### Body Composition

ADT is associated with a decrease in lean body mass (LBM) and increase in fat mass (FM), resulting in sarcopenic obesity [20]. These changes occur rapidly, starting after just 3 months of ADT [21, 22], with the average duration of therapy in high risk prostate cancer being 18 months [22]. After 1 year, FM has been shown to increase by 7–10%, while LBM has been shown to decrease by 2–4% [23], ten times the annual loss occurring in aging [24]. These changes are sustained up to 2 years after initiating ADT [25]. Hamilton et al. found that ADT results in accumulation of both visceral (22%) and subcutaneous (13%) fat, with increased insulin resistance likely arising from visceral fat accumulation [26].

The consequences of sarcopenic obesity in men with prostate cancer are significant. Cheung et al. reported that long-term ADT was associated with a reduction in lower-limb muscle function. The muscle groups most affected are those involved in generating body-weight support and regulating gait and balance [27]. This leads to increased frailty, with a cross-sectional study showing that between 22 and 24% of current and past ADT users were recurrent fallers, compared to 5% of men not on ADT [28]. These falls were also more likely to result in injuries including haematomas and fractures [28].

Weight gain after a prostate cancer diagnosis is associated with poorer outcomes [29]. A higher baseline BMI correlates with greater prostate cancer specific mortality (PCSM) [30, 31] and obesity is associated with higher rates of biochemical recurrence after prostatectomy for early stage prostate cancer [32]. Furthermore, a meta-analysis of prospective cohort studies reported a 15% higher risk of PCSM per 5 kg/m^2^ increase in BMI [33].

### Insulin Resistance and Diabetes Mellitus

Insulin resistance and type 2 diabetes mellitus are known complications of ADT. Multiple prospective studies have shown decreased insulin sensitivity during ADT with a 25.9% increase in fasting plasma insulin levels and 12.8% reduction in insulin sensitivity after just 3 months [34]. After 1 year of ADT, insulin resistance as measured by HOMA-IR increased by 39% [35].

Multiple studies have also consistently reported a significant link between ADT and subsequent diagnosis of diabetes [36–38]. In a retrospective study of 12,191 men with prostate cancer, ADT was associated with a 60% increased risk of diabetes [39]. These changes in glucose metabolism occur before any changes in body composition are apparent, highlighting the direct effect of ADT on glucose metabolism.

Higher insulin and glucose levels are associated with a worse prostate cancer prognosis [40]. Higher c-peptide levels (surrogate marker for endogenous insulin production) are associated with increased risk of PCSM as well as high-risk prostate cancer (Gleason ≥ 7) [41, 42]. Similarly, a meta-analysis of 17 cohort studies showed that pre-existing diabetes was associated with a 29% increase in PCSM and 37% increase in all-cause mortality in prostate cancer patients [43].

### Growth Factors and IGF-Binding Proteins

There are many alterations in hormonal, metabolic and inflammatory pathways in response to ADT that may contribute to the development of diabetes and insulin resistance. Insulin-like growth factor-1 (IGF-1) is a peptide produced by the liver and is involved in regulation of cell proliferation and differentiation. IGF-1 exerts multiple effects on glucose, fat and protein metabolism. The production of IGF-1 is stimulated by growth hormone (GH) secretion from the anterior pituitary gland which is potentiated by testosterone [44]. ADT has been shown to have either no effect on circulating IGF-1 or a 10% increase after 6 months of combined anti-androgen and GnRH therapy [45, 46]. Higher serum levels of IGF-1 are associated with increased all-cause mortality and PCSM in men with advanced prostate cancer [47]. These detrimental effects are also seen in studies of prostate cancer xenografts, where increased expression of IGF-1 and its receptor by prostate cancer cells results in tumour progression to castrate resistant prostate cancer (CRPC) [48].

The actions of the IGFs are modulated by a family of high-affinity IGF binding proteins (IGFBPs 1–6) which function to regulate IGF-1 and IGF-2 bioactivity [49]. IGFBP-2 is the main IGFBP produced by prostate epithelial cells, and is increased in patients with prostate cancer, correlating with tumour stage and grade [50]. Following androgen withdrawal, higher IGFBP-2 mRNA expression promotes androgen-independent tumour growth, and also correlates with a higher Gleason score [51, 52]. Conversely, higher serum IGFBP-3 is associated with a lower risk of developing advanced-stage prostate cancer [51, 53] and studies show an increase in IGFBP-3 beginning within months of androgen withdrawal [54]. As IGFBP-3 is the principal binding protein for IGF-1, an increase in IGFBP-3 is expected to reduce IGF-1 bioavailability. Thus, higher circulating IGFBP-3 would be of great advantage in cancer patients, exerting direct effects on cancer cells as well as reducing IGF bioactivity.

### Lipid Profile

ADT is associated with altered lipid metabolism. After 12 months of ADT, Smith et al. [55] found a 9.0% increase in total cholesterol, 11.3% increase in high-density lipoprotein (HDL), 26.5% increase in triglycerides and 7.3% increase in low-density lipoprotein (LDL). Like changes in body composition, these changes are rapid and can occur as early as 3 months following initiation of ADT [56].

Current evidence strongly suggests that lipid availability to cancer cells, whether newly synthesized or exogenously acquired, likely promotes prostate cancer growth and progression [57]. Elevated serum triglycerides are associated with increased risk of prostate cancer recurrence after a radical prostatectomy [58]. Similarly, high total cholesterol correlates with increased risk of lymph node metastases and high LDL levels are predictive of high Gleason scores [59]. Furthermore, elevated lipids, along with the aforementioned metabolic changes, also result in high cardiovascular mortality amongst prostate cancer patients receiving ADT [60].

### Cardiovascular Disease

Cardiovascular disease (CVD) accounts for approximately a quarter of deaths amongst men with prostate cancer [61]. ADT may indirectly contribute to development of CVD by inducing metabolic changes that are well-established risk factors for development of atherosclerosis [62]. In addition, ADT interferes with the cardioprotective property of testosterone, increasing the risk of adverse events [63]. GnRH agonist-mediated immune activation has also been linked to CVD via fibrous cap disruption and plaque destabilisation by activated circulating T cells with the capacity to express the GnRH receptor [63]. There is clinical evidence which suggests a positive association between ADT and CVD [62]. A meta-analysis of six observational studies showed that the risk of cardiovascular mortality was 17% higher amongst those receiving ADT than those not receiving ADT [60]. O’Farrell et al. [63] found the highest risk of mortality in those with a history of CVD before cancer diagnosis, and in the first 6 months of ADT. For these reasons, the United States Food and Drug Administration has issued a warning on GnRH agonists for increased risk of diabetes and certain CVDs (heart attack, sudden cardiac death and stroke) [64].

### Changes in Other Hormonal Systems, Myokines Inflammatory Cytokines

Circulating adipokines such as adiponectin and leptin are important regulators of insulin sensitivity. Prostate cancer patients undergoing ADT have leptin levels double that of those who have just undergone prostatectomy and/or radiotherapy without ADT [65]. Leptin levels increase in proportion to increase in fat mass, especially central adiposity. Studies of leptin levels and prostate cancer aggressiveness have produced mixed results. While some studies show a positive association between leptin levels and Gleason score [66, 67], others did not find serum leptin to be a predictive biomarker for advanced stage following radical prostatectomy [68].

Housa et al. [69] found higher adiponectin levels in locally advanced, compared to organ confined prostate cancer, and proposed that increased serum adiponectin levels may serve as a protective factor against tumour progression. Conversely, other studies found a negative association between plasma adiponectin levels and histological grade and stage [68, 70]. Levels of adiponectin have been found to increase with ADT [71, 72]. This is a paradoxical finding, as generally, adiponectin is characterised by a strong inverse correlation with fat mass and insulin resistance [71]. However, the increase in adiponectin does not seem sufficient to counteract the adverse effects of ADT on hyperinsulinaemia [73].

Pro-inflammatory cytokines have also been implicated in the development of diabetes and may be modulated by testosterone [74]. After 12 weeks of ADT, there is a fall in interleukin 6 (IL-6) levels along with higher levels of interleukin 1 beta (IL-1β) and interleukin-8 (IL-8) [75]. Conversely, Maggio et al. found that 12 months of ADT did not affect plasma cytokine levels in men with prostate cancer [76]. Obesity is associated with a subclinical inflammatory state with higher plasma concentrations of pro-inflammatory mediators such as IL-6, tumour necrosis factor-alpha (TNF-α) and IL-1β [77]. Based on epidemiological studies, higher IL-6 levels are associated with prostate cancer biochemical recurrence [78] and poorer overall survival [79]. Increased serum IL-6 levels are also found in patients with castrate-resistant and metastatic prostate cancer [79, 80]. Similarly, higher levels of TNF-α are associated with more aggressive disease, prostate cancer progression, relapse and mortality [75, 81, 82].

### Effect on Bone Mineral Density

ADT is associated with a significant reduction in bone mineral density (BMD), with more rapid bone loss compared to normal aging. Post-menopausal women experience an annual average of 3% decline in BMD at the spine [83]. Following initiation of ADT, the annual rates of bone loss at the lumbar spine and femoral neck regions have been reported as 4.6% and 3.8%, respectively [84]. In a cross-sectional study, men with prostate cancer treated with ADT had a 7.2–7.8% lower lumbar spine BMD, and trends towards a lower hip BMD compared to men not receiving ADT and healthy controls [85]. This reduction in BMD is translated into a higher fracture risk. In a large cohort study of 180,000 older men, ADT increased the relative risk of any fracture and hip fracture by 1.4 [86], thus increasing morbidity and mortality.

### Psychophysiological Effects

The prevalence of depression and anxiety in men with prostate cancer across the treatment spectrum is high [87]. In particular, men receiving ADT have clinically significant decreased quality of life, particularly in the physical and sexual aspects compared to controls [88]. These psychological conditions are associated with psychophysiological side effects that encompass poorer treatment outcomes and reduced survival [87, 89, 90]. In turn, depression results in a chronically activated hypothalamo-pituitary-adrenal axis, immune dysfunction, inflammation, oxidative stress and increased cytokine production thus worsening cancer prognosis [91].

### Summary

In summary, the negative systemic effects of ADT can potentially worsen prostate cancer prognosis. In the next section of this review, we discuss the clinical trials that utilise PRT in the treatment of these effects in prostate cancer.

## The Benefits of PRT During ADT

### PRT and Physiological Adaptations

Muscle hypertrophy induced by PRT is the product of increased muscle fiber cross-sectional area [92] and is accompanied by the enhancement of subcellular structures (e.g. mitochondrial morphology and density) and increased substrate metabolism. This improvement in the metabolic capacity of skeletal muscle underlies a range of beneficial adaptations that may be particularly important to men treated with ADT.

Much of the current evidence regarding muscle adaptation in PRT is drawn from studies involving the elderly population with sarcopenia, a similar cohort to those on ADT [93]. In sarcopenia, there is a reduction in the number of both slow-twitch type I and fast-twitch type II muscle fibers and specific type 2 muscle fiber atrophy [94], leading to a decline in muscle strength [95]. PRT in this population has been shown to increase type IIa muscle fiber cross-sectional area [94, 96]. Thus, this physiological adaptation may improve physical function and contribute to improved glucose metabolism due to increased GLUT4 activity and enhanced insulin response via skeletal muscle [97]. Furthermore, PRT also has beneficial effects on mitochondrial function and proteostasis, the loss of which is implicated in the pathophysiology of muscle loss in sarcopenia [98].

### PRT in the Treatment of ADT-Induced Adverse Effects

The benefits of isolated PRT in the treatment of ADT-induced adverse effects have been shown in five randomized controlled trials to date [99–103]. The details of each trial, including sample size, duration, type of intervention and findings, are summarized in Tables 1 and 2.

**Table 1:** Study characteristics of the five included RCTs

Study identification	Population		Sample size (N) Mean age (year)	PRT intervention	Control condition	Duration (weeks)
	Major inclusion criteria	Major exclusion criteria				
Alberga et al. 2012 Canada [99]	Histologically documented prostate cancer; scheduled to receive radiotherapy with or without ADT; consent of treating oncologist (Note: this article reported a subgroup analysis limited to patients on ADT)	Severe cardiac disease (New York Heart Association class III or IV); uncontrolled hypertension, pain, psychiatric illness; residence > 1 h from the study center.	*N* = 49 66 year	PRT supervised by a certified fitness consultant, 3 sessions/week, 24 weeks. Ten exercises (i.e. leg extension, leg curl, seated chest fly, latissimus pulldown, overhead press, triceps extension, biceps curls, calf raises, low back extension, and modified curl-ups) using 60-70% 1RM load, 8-12 reps per set, 2 sets per exercise. Load increased by 5lb (2.3kg) when able to complete > 12 reps/set.	Usual care (no exercise)	24
Nilsen et al. 2015, 2016 Norway [100, 104]	Intermediate or high-risk profile based on PSA and histology and extent of the primary tumour; referred to high-dose radiotherapy 2–6 months after initiation of neo-adjuvant ADT; adjuvant ADT continuing for 9-36 mo.; age ≤75 years;	Strength training ≥ 1 session/week; osteoporosis medication usage; medical conditions that could complicate participation	*N* = 58 66y	Two supervised (high intensity) plus one unsupervised (moderate intensity) PRT session/wk, 16 weeks. Nine exercises (i.e. Smith machine half squat, leg press, Smith machine standing calf raises, knee flexion, knee extension, chest press, seated row, seated shoulder press, and biceps curl), 1–3 sets/exercise, 6–10 RM, loading increased with strength adaptation	Usual care (no exercise)	16
Segal et al. 2003 Canada [101]	Histologically documented prostate cancer; scheduled to receive >3mo. ADT; consent of treating oncologist	Severe cardiac disease (New York Heart Association class III or IV); uncontrolled hypertension (> 160/95 mmHg); uncontrolled pain; unstable bone lesions; residence > 1 h from the study center.	*N* = 135 68y	PRT supervised by a certified fitness consultant, 3 sessions/week, 12 weeks. Nine exercises (i.e. leg extension, calf raises, leg curl, chest press, lat pulldown, overhead press, triceps extension, biceps curls, and modified curl-ups) using 60–70% 1RM load, 8–12 reps per set, 2 sets per exercise. Load increased by 5 lb (2.3 kg) when able to complete > 12 reps/set.	Usual care (no exercise)	12
Taafe et al. 2017 Australia [102]	Histologically confirmed prostate cancer; > 2-month exposure to and anticipated to receive 12 months additional ADT; without PSA evidence of disease activity; medical clearance	Bone metastatic disease; evidence of PSA disease activity; chronic conditions that could inhibit exercising; non-ambulatory; structured exercise in the previous 3 months;	*N* = 105 68 year	Two supervised PRT group-based sessions (*n* < 10), 2 sessions/week, 52 weeks. Impact-loading: bounding, skipping, drop jumping, hopping, and leaping activities that produced ground reaction forces of 3.4–5.2 times body weight and progressive in nature. Resistance training: six principal exercises (i.e. chest press, seated row, shoulder press, leg press, leg extension, and leg curl) and supplementary exercises, 2–4 sets per exercise at an intensity of 6–12 RM. Participants also performed impact loading routine 2 sessions/week at home.	Usual care (no exercise)	52
Winters-Stone et al. 2014, 2015 USA [105]	Histologic evidence of prostate cancer; currently receiving ADT; BMD T-score − 2.5 or higher; medical clearance from physician	Currently receiving chemotherapy; bone metastases in the hip or spine, bone-active medications other than ADT (e.g., bisphosphonates); participation in 30 min or more of moderate–vigorous PRT per week	*N* = 51 70 year	Two supervised plus one home-based PRT session/week, 52 weeks. PRT exercises (i.e. wall-sits, squats, bent-knee deadlifts, lunges, one-arm row, chest press, lateral raise, and push-ups) and impact loading (i.e. two-footed jump) using free weights or weighted vest. PRT performed for 1–2 sets x 6–14 reps/exercise. Jumping progressed from 1–10 sets x 10 reps. All loading progressed with strength gains.	Placebo control (stretching and relaxation)	52

**Table 2:** Key results of the five included RCTs

Study identification	Physiological outcomes (assessments, units)	Adherence to PRT intervention	Key results
Alberga et al. 2012 Canada [99]	Body weight (kg); BMI (kg/m^2^); DEXA (total lean mass (kg) and percent body fat (%))	Not reported	Percent body fat increased in the control group versus PRT group (*p* = 0.005); total lean mass was maintained in the PRT group versus a loss in the control group (*p* = 0.005)
Nilsen et al. 2015, 2016 Norway [100, 104]	Body composition via DEXA (lean body mass: total, trunk, lower extremities, upper extremities, appendicular; fat mass: total and trunk in kg, and percent body fat (%)); body mass (kg); BMI (kg/m^2^); BMD (total body, total lumbar, total hip, trochanter, femoral neck); skeletal muscle biopsy (total muscle CSA, type I and II muscle CSA, μm^2^; number of myonuclei, nuclei/fiber; myonuclear domain, cytoplasm:nuclei; number of satellite cells; androgen receptors, myostatin, mitochondrial proteins (i.e. citrate synthase, cytochrome c oxidase subunit IV (COXIV), HSP60); indicators of muscle cellular stress (HSP70, alpha B-crystallin, HSP27, free ubiquitin, and total ubiquitinated proteins)	84% and 88% upper and lower body exercises completed, respectively	Lower extremity (*p* = 0.01), upper extremity (*p* = 0.05) and appendicular (*p* = 0.001) lean body mass significantly increased in PRT versus control. No change in BMD outcomes. Significant increase in total muscle fiber CSA in PRT versus control (*p* = 0.04) showing a larger effect in type II (*p* = 0.03) than type I fibers (*p* = 0.11). Significant increase in number of myonuclei per type I fibers (+ 17%, *p* = 0.01) but not type II fibers in PRT versus control. Significant reduction in myonuclear domain in type I fibers but not type II fibers in PRT versus control (*p* = 0.05). No change androgen receptor or myostatin content, or any mitochondrial protein or indicator of muscle cellular stress. Post hoc: within-group analysis revealed that HSP70 was reduced in the PRT group and a trend towards a reduction in citrate synthase in the control group
Segal et al., 2003 Canada [101]	Body weight (kg); BMI (kg/m^2^); waist circumference (cm), sum of four skinfolds (mm)	79%	No change in body weight, BMI, waist circumference or subcutaneous skinfolds
Taafe et al. 2017 Australia [102]	PSA (ng/ml), total testosterone (ng/dl)	69%	No significant change in PSA or testosterone
Winters-Stone et al. 2014, 2015 USA [103, 105]	BMD of proximal femur (total hip, greater trochanter, and femoral neck) and lumbar spine (L1-L4) via DEXA; bone turnover via serum osteocalcin (ng/mL) and urinary deoxypyrodinoline (nmol/L); Body composition via DEXA (total lean mass, total fat mass, and trunk fat mass in kg; percent body fat (%)); insulin (mclU/ml); IGF-1 (ng/ml); SHBG (nmol/ml); total testosterone (ng/dl); body weight (kg)	84% and 43% for supervised and home-based sessions, respectively	PRT had a significant effect on preservation of BMD (− 0.4%) at the L4 vertebrae compared with losses (− 3.1%) in the placebo control group (*p* = 0.03). Adjusting for patients who completed the study (i.e. follow-up assessments) the PRT program significantly reduced total fat mass (*p* = 0.02) with a trend towards reduced body fat percentage (*p* = 0.06) and trunk fat mass (*p* = 0.07) versus control; and deoxypyrodinoline decreased in the control group versus the PRT group (*p* = 0.03). Reduction of total fat mass (*p* = 0.04) and trunk fat mass (*p* = 0.03) associated with reductions in fasting insulin

#### Effect on Body Composition and Muscular Strength

PRT has been shown to be beneficial in the maintenance of LBM during ADT. Alberga et al. [99] found that patients randomized to the PRT group was able to maintain total LBM as versus the control group. Similarly, Nilsen et al. [100] documented a site-specific increase in LBM of the lower limb, upper limb and appendicular region in patients receiving 16 weeks of PRT versus control. Skeletal muscle biopsies were collected in one trial [100]. Patients in the PRT group had a significant increase in total muscle fiber cross-sectional area, with the greatest effect noted in type II muscle fibers, as versus those in the control group, who had an overall reduction [104]. The number of myonuclei per type 1 fiber also increased in the PRT group.

There was a consistent improvement in both upper and lower arm strength across four trials following 3 to 12 months of PRT [99–102]. Taafe et al. [102] also reported an improvement in cardiorespiratory fitness in the PRT group, as reflected by an increase in the 400 m walk test.

PRT also has positive effects on FM. Alberga et al. [99] reported that percent body fat significantly increased in the control group versus the PRT group after 24 weeks. Likewise, Winters-Stone et al. reported a reduction in FM in patients undergoing PRT, as opposed to the control group who gained fat mass [103].

#### Insulin Resistance and Type 2 Diabetes

The effect of PRT in men on ADT has not been extensively evaluated. Only one trial by Winters-Stone et al. [103] reported a reduction in serum insulin and IGF-1 levels in the PRT group compared to an increase in both biomarkers in the control group.

#### Bone Mineral Density

Only two trials investigated bone mineral density (BMD) changes following 16 [100] and 52 [105] weeks of PRT. No differences in BMD outcomes were noted except preservation of BMD at the L4 site in patients in the PRT group versus the control group [105]. Bone turnover markers including osteocalcin and urinary deoxypyridinoline did not change [105].

#### Psychological Effects

Physical exercise is recognised as a powerful modulator of neuroplasticity and immune response with immunosurveillance-enhancing properties [106]. Health-related quality of life (HRQOL) was assessed in three studies [100–102]. Segal et al. [101] reported an improvement in HRQOL following PRT while no differences were found by Nilsen et al. [100]. Taafe et al. [102] found improvement in fatigue and vitality after 6 and 12 months of PRT.

### Summary

In summary, PRT is beneficial in the treatment of ADT-induced adverse effects, with positive effects on body composition, muscle strength and cardiorespiratory fitness and QOL. However, there is currently inconclusive evidence establishing the relationship between PRT and prostate cancer progression and recurrence.

## The Effect of PRT on Cancer Growth Pathways

**Fig. 1:**
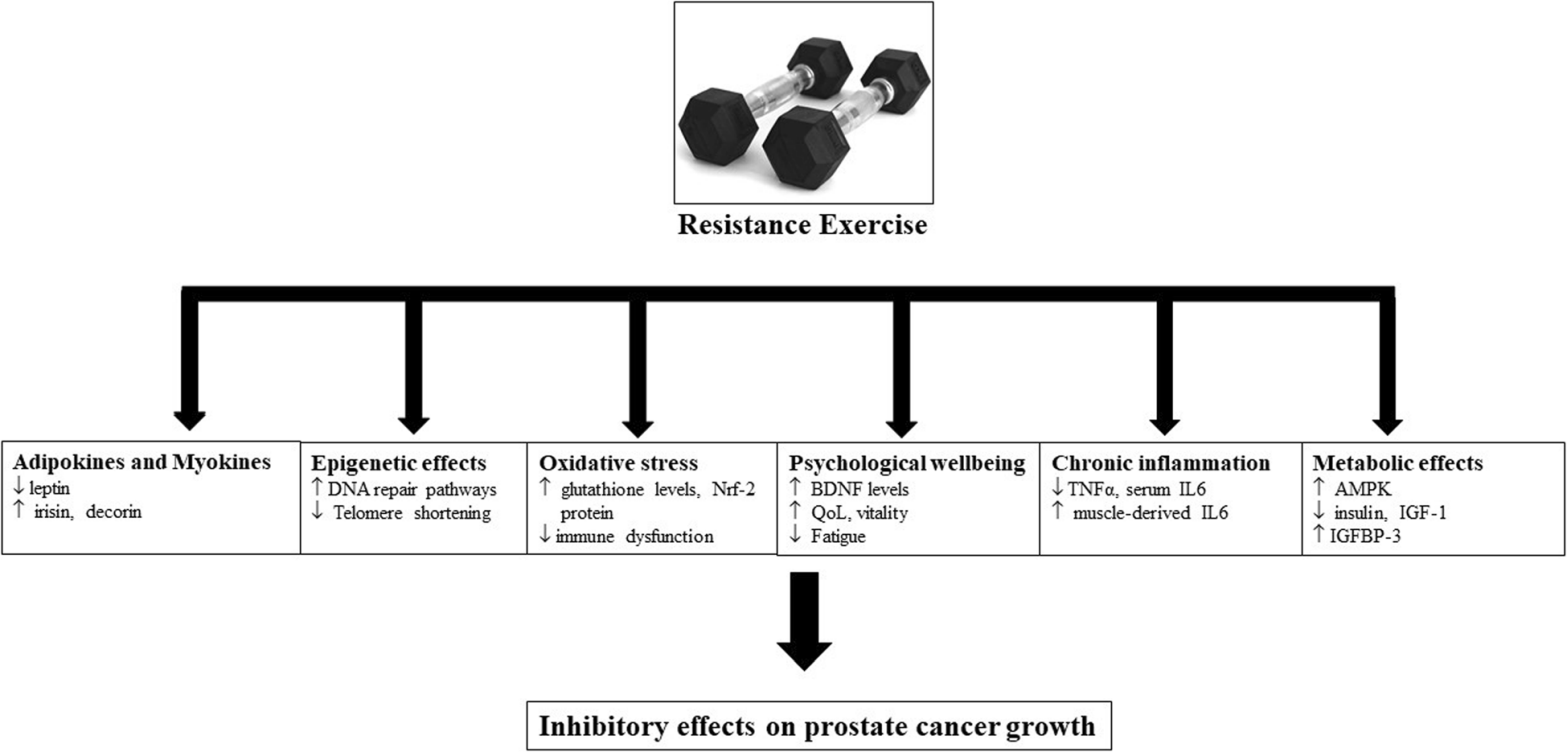
The potential inhibitory effects of resistance training on the prostate cancer growth pathway

In a prospective cohort study following over 2000 men with prostate cancer, it was found that men who were physically active lived significantly longer. Three or more hours per week of vigorous exercise was associated with a 61% decreased risk of dying from prostate cancer [107]. Although this association does not necessarily indicate causation, it has led to interest in exploring mechanisms by which exercise might favourably influence the biology of cancer cell growth. While it is known that exercise can lower the risk of developing cancer, and is associated with lower relapse rates and increased survival, its precise anti-cancer effects have not been fully established [89]. In the above, the important role of PRT in the treatment of the adverse effects of ADT was discussed. The next section provides a summary of current literature regarding the potential benefits PRT has on oncogenic pathways in prostate cancer. As there is currently a paucity of studies in this area, we also incorporate evidence derived from studies of other pathologies and cancer types. This is outlined in Fig. 1.

### Metabolic Effects

Muscle tissue produces many factors that are associated with cancer progression and metastatic potential. When muscle contraction occurs during exercise, adenosine triphosphate (ATP) is consumed for energy derivation, reducing the adenosine monophosphate (ATP/AMP) ratio. This results in cellular activation of the liver kinase B1- (LKB1-) adenosine monophosphate-activated protein kinase (AMPK) pathway. AMPK inhibits the mammalian target of rapamycin (mTOR) protein, which has been implicated in prostate cancer progression [108]. Powerful muscle contraction results in potent stimulation of AMPK [109] which also results in translocation of the GLUT-4 membrane transporter in myocytes [110], leading to glucose influx and lowering of serum glucose levels, which has a favourable impact on prostate cancer prognosis [111]. Stimulation of AMPK also suppresses tumour growth, uptake of glucose and aerobic glycolysis of tumour cells, known as the Warburg effect [112].

Both insulin and IGF-1 regulate cell proliferation, differentiation, survival and apoptosis. These molecules bind to their tyrosine kinase receptors and activate several signalling pathways including phosphoinositide 3-kinase (PI3K)/protein kinase B (AKT)/mTOR resulting in inhibition of apoptosis and promotion of cell growth and angiogenesis [113]. The principal binding protein of IGF-1, IGFBP-3, can reduce IGF-1 bioactivity further inhibiting cancer growth [114]. IGFBPs not only modulate the bioavailability and signalling of IGFs but also have independent actions on cell growth and survival [49]. In-vitro studies have shown IGFBP-3 to inhibit proliferation, adhesion, invasion and metastasis of prostate cancer, independent of IGF-1 [115, 116]. IGFBP-3 is also a potent inhibitor of MAPK signalling, which is implicated in the development of castrate-resistant prostate cancer [117]. Higher serum IGFBP-3 is associated with a lower risk of developing advanced-stage prostate cancer [53]. However, while PRT has been shown to reduce plasma IGF-1 [103] and increase IGFBP-3 [114] levels in prostate cancer, current epidemiological studies in cancer populations show significant heterogeneity in the response of the systemic IGF axis to exercise [118]. This discrepancy may be attributed to baseline concentrations of the IGF ligands, as Nishida et al. [119] showed that participants with elevated baseline IGF-1 experienced the greatest decrease in response to exercise. Furthermore, there are current limitations in oncological research regarding the exercise response of autocrine, as compared to systemic IGF-1 [118]. In older adults with rheumatoid arthritis, PRT increased total lean and appendicular muscle mass, which was associated with increases in muscular IGF-1 and IGFBP-3 with no changes in systemic levels [120]. Thus, more studies are required on the specific tissue response of the IGF-1 axis to PRT in prostate cancer.

### Chronic Inflammation and Antioxidant Pathways

It is known that chronic inflammation in prostate cancer is associated with prostate cancer progression and poorer overall survival [81]. Stimulation of muscle contraction during PRT releases myokines that lower systemic inflammation [121], with 4–8 weeks of PRT reducing serum IL-6 and TNFα in prostate cancer patients [122]. While IL-6 derived from macrophages and adipocytes has pro-inflammatory effects [121], muscle-derived IL-6 can counteract TNF-α, which as previously discussed, is associated with significantly worse outcomes in men with prostate cancer [81]. Muscle-derived IL-6 acts as an energy sensor, and improves overall metabolic function by increasing insulin-stimulated glucose uptake and whole-body fatty acid oxidation [121]. Thus, modulation of systemic inflammation may represent one of the pathways in which PRT may inhibit prostate cancer progression.

Skeletal muscle is a major source of reactive oxygen species (ROS) which are balanced by antioxidant enzymes such as catalase, glutathione peroxidase and glutathione reductase [123]. ROS increase oxidative stress on DNA, which can contribute to the initiation and progression of prostate cancer [124]. In healthy young men, resistance exercise performed regularly for 6 weeks decreased oxidative stress and increased glutathione levels [125]. Prostate cancer patients who participated in vigorous activity had greater expression of the nuclear factor erythroid 2-related factor 2 (Nrf-2) in their normal prostate tissue compared to those who were more sedentary. The Nrf-2 protein stimulates the production of anti-oxidant enzymes, and studies in mice show that loss of Nrf-2 correlates with increased ROS and DNA damage leading to neoplastic transformation of normal prostate tissue [126].

### Adipokines and Myokines

Leptin is an adipokine which is a key regulator of appetite control and body weight. It also has a role in energy homeostasis, insulin secretion, angiogenesis and modulation of innate and adaptive immune responses [127]. High circulating levels of leptin enhance growth of prostate cancer cells in vitro [128] and PRT has been found to significantly reduce serum leptin levels in obese men [129]. Resistin is an adipokine known to upregulate pro-inflammatory cytokines [130], and induce prostate cancer cell proliferation [131] while adiponectin has anti-inflammatory properties [132]. PRT has been found to reduce serum resistin levels in post-menopausal women [133] while increasing serum adiponectin in obese young men [134], but has not been extensively studied in the cancer population.

Irisin is a myokine generated in the presence of exercise-induced upregulation of peroxisome proliferator-activated receptor gamma coactivator-1-alpha (PGC-1α). It has a role in the regulation of energy metabolism, browning of white adipocytes and improving insulin sensitivity [135]. Irisin has been shown to significantly reduce cancer cell proliferation, migration and viability in malignant cancer cell lines without affecting non-malignant cells. Specifically, irisin has cytotoxic effects on prostate cancer cells [136]. It has been suggested that endurance training can increase circulating irisin levels in human subjects [137]. Similarly, Zhao et al. [138] found that 12 weeks of PRT significantly increased serum irisin in older adults. Another potential pathway may involve decorin, which is a proteoglycan and a myokine, stimulated by resistance training [139]. Recent discoveries show that decorin reduces cancer growth and dissemination [140]. In prostate cancer cell models, decorin prevents androgen receptor nuclear translocation and inhibits the production of PSA [141]. In an animal model, systemic administration of decorin significantly reduced prostate cancer bone metastasis [142]. Thus, myokines released during muscle contraction may have a direct effect reducing cancer growth and spread.

### Neurotrophic Pathway

Brain-derived neurotrophic factor (BDNF) is a member of the neurotrophin family of growth factors which supports differentiation, maturation and survival of neurons in the nervous system and a reduction in BDNF is implicated in the development of depression [143]. BDNF is also secreted by prostate cancer cells and has mitogenic effects on the prostatic epithelium [144]. A 12-week resistance training program in older male subjects was found to increase circulating plasma BDNF levels which returned to baseline after de-training [145]. Thus, the BDNF signalling pathway may represent one of the modalities by which resistance exercise inhibits prostate cancer cell growth.

### Epigenetic Effects

Exercise has epigenetic effects on the phenotypic expression of various genes involved in cancer [89]. In men with low risk prostate cancer, cell cycling and DNA repair pathways were upregulated in those who participated in ≥ 3 h/week of vigorous activity compared to those who did not [146]. MicroRNAs (MiRNA) are small, endogenous non-coding RNA which can modify protein expression through cleavage of specific target mRNAs or through inhibition of their translation. In prostate cancer, the presence of oncogenic miRNAs such as miR-21 is predictive of cancer recurrence following radical prostatectomy [147], and serum levels of miR-21 have been found to decrease immediately after resistance exercise in healthy young men [148]. Dimauro et al. [149] found that 12 weeks of moderate intensity, explosive-type resistance training in an elderly cohort counteracts shortening of telomeres, which are nucleotides at the end of chromosomes that protect their integrity. Telomere shortening is one of the earliest molecular genomic events in prostate tumorigenesis and can generate genomic instability [150]. In men with early prostate cancer, those who followed a comprehensive lifestyle program, including regular exercise, had increases in telomere length after 5 years [151].

## Future Research Directions and Conclusion

To conclude, as the population ages and the number of prostate cancer diagnoses increases across the population, we are likely to encounter more of the deleterious effects of ADT. There is now an endorsement by various oncological societies to incorporate the use of physical activity as synergistic medicine during prostate cancer treatment. PRT is an exercise modality that has been shown to be of benefit in the maintenance of body composition and muscle function during ADT, although it is important to note that current evidence exhibits major heterogeneity within and between studies in terms of patient characteristics and type of PRT intervention. Despite compelling evidence for the application of PRT as standard of care in patients with prostate cancer, there is still paucity in the literature regarding its use in this population. This includes its benefits on specific muscle groups, and its impact on physiological endpoints such as glucose and insulin metabolism, bone turnover and the adipokines, myokines and inflammatory cytokines affected during ADT, thus providing scope for future research.

Furthermore, this review summarises the current body of evidence on the potential signalling pathways modified either directly or indirectly by PRT, and its positive effects on cancer growth and progression. As many of these pathways are also implicated in the development and progression of prostate cancer, more clinical studies are required in this area to obtain a better understanding of the mechanisms of benefit of PRT.

In summary, PRT is an exercise modality with great potential in the treatment of ADT-induced adverse effects. However, more research is required regarding its impact on the physiological and biochemical pathways involved in prostate cancer progression.

## Data Availability

Not applicable.

## Abbreviations

ADT: Androgen deprivation therapy
AMP: Adenosine monophosphate
AMPK: Adenosine monophosphate-activated protein kinase
ATP: Adenosine triphosphate
BDNF: Brain-derived neurotrophic factor
BMD: Bone mineral density
CRPC: Castrate resistant prostate cancer
FM: Fat mass
GH: Growth hormone
GnRH: Gonadotrophin-releasing hormone
HbA1c: Glycosylated haemoglobin
HDL: High-density lipoprotein
HRQOL: Health-related quality of life
IGF-1: Insulin-like growth factor-1
IGFBP: IGF binding protein
IL-1β: Interleukin 1 beta
IL-6: Interleukin 6
IL-8: Interleukin 8
LBM: Lean body mass
LDL: Low-density lipoprotein
MiRNA: MicroRNAs
mTOR: Mammalian target of rapamycin
Nrf-2: Nuclear factor erythroid 2-related factor 2
PCSM: Prostate cancer specific mortality
PGC-1α: Peroxisome proliferator-activated receptor gamma coactivator-1-alpha
PI3K: Phosphoinositide 3-kinase
PSA: Prostate-specific antigen
TNF-α: Tumour necrosis factor alpha
